# The Effect of Biodegradable Waste Pyrolysis Temperatures on Selected Biochar Properties

**DOI:** 10.3390/ma14071644

**Published:** 2021-03-27

**Authors:** Katarzyna Wystalska, Anna Kwarciak-Kozłowska

**Affiliations:** Faculty of Infrastructure and Environment, Czestochowa University of Technology, 42-200 Czestochowa, Poland; anna.kwarciak@pcz.pl

**Keywords:** biodegradable waste, biochar, circular economy, pyrolysis temperature

## Abstract

Biochars produced during biodegradable waste pyrolysis are products with a wide range of environmental applications. The effect of impact biochars depends on their properties which determine the course of specific processes. The main aim of the study was to investigate the effect of pyrolysis temperature on selected properties of biochar produced from various plant wastes (beech wood chips, walnut shells, wheat-rye straw), the valorization of which is of key importance for the implementation of the circular economy. Biochars were produced at temperatures of: 400 °C, 500 °C, 600 °C and 700 °C in a nitrogen atmosphere. An increase in the pyrolysis temperature caused a drop in the biochar production yield. As the temperature increased, higher carbon content and lower hydrogen content could be seen in the products obtained. An increase in the pH and total organic carbon (TOC) values also found. The influence of temperature on ash content, observed in the case of BWS (biochar from walnut shell) and BWRS (biochar from wheat and rye straw), did not occur in the case of BWC (biochar from beech wood chips). Another parameter that demonstrated a growing tendency with increasing temperature was the BET specific surface area (except for biochars from wheat and rye straw). An increase in pyrolysis temperature caused a decrease in the diversity and density of the surface functional groups of biochars. The influence of the type of precursor used in the production of biochar on the presence of surface functional groups was demonstrated. The presence of intense stretching vibrations of C–O bonds, having a potential impact on the sorption capacity of biochars, was determined in the FTIR spectra of BWC600 and BWC700 biochars, this feature, combined with the large BET surface area, may affect the sorption potential of these biochars. The presence of this type of high-intensity vibrations was also observed in the spectra of biochar BWRS600 and BWRS700. This can compensate for the low BET surface value and play an important role when using these biochars in sorption processes for organic and inorganic compounds.

## 1. Introduction

One of the challenges of the circular economy, i.e., an economy which aims, among others, at minimizing the quantities of waste generated, is the capacity of recovering useful products from waste materials. Such a solution requires not only systemic transformations and changes in management, but also the development of new environment-friendly waste treatment technologies. This manner of waste handling makes it possible to reduce the use of natural resources and to generate energy and new materials. These actions are very significant for climate change mitigation and are therefore increasingly desirable for society.

Residues from agri-food production or wastes generated in the municipal sector continue to pose a significant problem for the managers of the economy regarding the choice of a method for their disposal. Appropriate waste management would not only eliminate their adverse environmental impacts (among others, greenhouse gas emissions, water and soil pollutants), but also, in line with the current expectations, enable new value added products to be derived [1,2,3,4,5]. These products could be incorporated into the environmental cycle while, at the same time, closing the nutrient cycles [6].

Waste treatment in the pyrolysis process corresponds well with the circular economy model. Pyrolysis is a well-known process that has long been used to treat different types of materials [7]. At present, it is increasingly often used to convert biodegradable wastes into biochars [1,7,8,9]. Biochars can be produced from biodegradable waste of both plant origin, e.g., straw, wood chips, nutshell, sawdust and sewage sludge and poultry manure [10,11,12,13,14]. Biochar production is maximized during slow pyrolysis [15], with a slow rate of temperature increase (0.01–2 °C·s^−1^) [16]. Slow pyrolysis can enhance phosphorus availability, which is favorable for the use of biochars [14,17,18] and is able to generate carbon abatement by the treatment of any type of waste [19]. Waste pyrolysis, especially high-temperature pyrolysis [20], can be an effective means of fixing metals in the process products, which can be of large importance for the treatment, among others, of sewage sludge often contaminated by heavy metals. The elements such as Fe, Zn, Mn and Cu are volatilized at temperatures higher than 700–800 °C [10]. The concentration of mineral nutrients is higher in the biochar produced at higher temperatures [10,21]. 

As demonstrated by multidirectional research, biochars can be used in a number of applications in environmental engineering or protection, among others, as an additive enhancing the “effectiveness of the composting” [22,23,24,25,26,27,28] (especially in the case of the treatment of substrates with substantial nitrogen content) or a material reducing greenhouse gas or odor emissions [11,29,30,31] which often occur during the composting process [32]. Biochars can be used as additives improving soil properties [33,34,35], reducing [36] the bioavailability of contaminants [37], enhancing plant growth and yield [33,34], and affecting the mobility of heavy metals [8,38,39,40,41,42] and their accumulation in plant biomass [43]. An extensively investigated application is the use of biochars as adsorbents of contaminants from different media [44,45,46,47]. 

The use and positive effect of biochars depend on its properties [10,48] and, in turn, these primarily result from the type of substrate used for its production, the pyrolysis temperature [10,48,49,50,51,52,53] and the heating rate [7,54]. The type of gas used in the pyrolysis process may affect properties of the obtained products [55]. The feedstocks for biochar production are characterized by a different elemental composition and lignocellulose content that affect the capacity of biochars to adsorb organic contaminants [11]. Therefore, in order to obtain a product with the expected properties, the type of feedstock and the parameters of the pyrolysis process have to be appropriately combined [8,36]. The pyrolysis temperature determines a number of properties of biochars and, as a result, their uses [48]. It affects the specific surface area, porosity, sorption capacity, ion exchange properties, pH, elemental composition, organic carbon content, presence of functional groups, stability of biochars and hydrophilicity of their surface [3,8,15,16,45,47,50,56,57,58,59,60,61,62]. 

The sorption capacity of biochars has been investigated by many authors in relation to both inorganic and organic substances [11,52,63,64,65,66]. Biochars can be used for the sorption of contaminants, among others, from aqueous solutions [47,65,67,68]. The capacity of biochars to adsorb different components makes them potential nutrient carriers, which are very desirable and attractive for environmental applications [6,12,68,69,70]. A large specific surface area, porosity, ion exchange capacity and thermal stability of biochars affect their sorption properties [69]. In addition to the specific surface, the presence of non-carbonized organic matter, hydrophobicity and aromaticity of biochars also affect the sorption of specific components [11,66]. The properties of the biochars described above are determined by the pyrolysis temperature. A higher temperature of this process can improve the sorption capacity of biochars [71]. However, a high biochar production temperature is not always correlated with high adsorption capacity [68,69,70]. This means that biochars produced at both lower and higher temperatures can be good sorbents depending on particular biochar properties and binding mechanisms typical for different chemical substances. The metal sorption could be the results of mechanisms based, among others, on electrostatic interactions, ion exchange, binding of metals by functional groups present on the surface of biochars or precipitation [58,72]. These mechanisms are determined by the properties of biochars prevailing in products derived at lower or higher temperatures [44]. The presence of organic carbon phases, rich in surface functional groups, in biochar contributes to alkaline pH, negative charge, cation exchange and the ability to form organometallic complexes. Mineral components can ensure metal sorption as a result of electrostatic attraction, ion exchange, surface complexation and precipitation of metal sediments by releasing soluble ions [72].

Thus, by controlling the pyrolysis temperature, biochar sorbents can be produced for different applications, and their attractiveness can be effectively enhanced by modifying chemical and physical processes or creating composites with other raw materials [2,46,64,69,73,74,75,76]. The use of sorbents from waste materials can be a more economical process, given the high costs of commercial sorbents [44].

As other authors have concluded [56], the research to determine the optimum conditions for biochar production from different raw materials continues to be necessary in order to identify a complete set of parameters that would enable biochar production for a specific application [36,56]. The research done to date [7,8,11,52,77] confirms the need to appropriately design the properties of biochars to ensure that their use in the environment brings the expected benefits.

The production of biochar sorbents can compete with commercial products that are much more expensive. It can contribute to reducing the amount of landfilled or incinerated waste, as a result of the production of added-value products that can be included in the technological cycle, e.g., wastewater treatment plants, biogas plants, composting plants, or landfills. Therefore, the main goal of our work was the valorization of selected types of waste, in terms of practical use, among others in sorption processes. The aim of the research was to show the effect of pyrolysis temperature on the properties of the produced biochars and to present the characteristics of biochars produced from various wastes. The novelty of the conducted research was the analysis of the correlation of the influence of the type of precursor and the temperature of the pyrolysis process on the properties of the produced biochars. The results of this analysis may prove helpful in designing the valorization process of selected types of waste in the pyrolysis process. They can help assess the suitability of the produced biochars for various applications and choose the method of modifying their properties.

## 2. Materials and Methods

### 2.1. Substrates for Biochar Production

Biodegradable wastes of plant origin, i.e., walnut shell (WS), beech wood chips (WC) and wheat and rye straw (WRS) were used for biochar production. The moisture content (MC), organic matter (OM) in dry mass, ash, pH, the Kjeldahl nitrogen content (N_K_) and the total carbon (TC) in dry mass—were determined for the particular substrates (Table 1). The highest total carbon content was found in beech wood chips and walnut shell, while a slightly lesser content of that component was determined for wheat and rye straw. Wheat and rye straw had the highest total nitrogen content at a level of about 0.5%. The determined pH was diversified for the particular substrates. Straw was characterized by a neutral reaction, while beech wood chips and walnut shell had a slightly acidic reaction. The water content in the substrates fell within the range of 7.19 to 13.38%.

### 2.2. Parameters of the Pyrolysis Process

**Figure 1:**
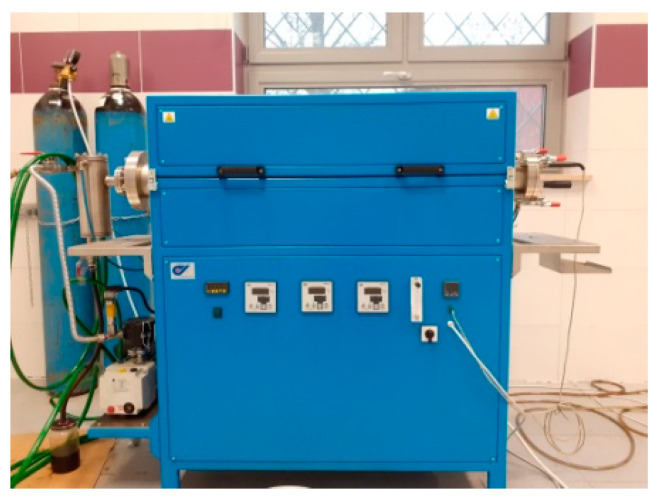
Pyrolysis reactor used for the production of biochar.

The substrates were subjected to thermal conversion in a pyrolysis reactor (PRW-S100 × 780/11 (Figure 1) in a nitrogen atmosphere (5 L·min^−1^). The pyrolysis reactor was manufactured by the Polish company Czylok (Jastrzębie-Zdrój, Poland) for the Czestochowa University of Technology. It is a tube furnace constructed on the basis of two-half heating and insulation modules. The heating temperatures of the substrate were: 400 °C, 500 °C, 600 °C and 700 °C. The heating times of samples were 120 min for temperatures 400 °C and 500 °C and 150 min for temperatures of 600 °C and 700 °C respectively. The retention time was 60 min. After the pyrolysis process was completed, the samples were left in the reactor until they reached room temperature. Biochar samples were stored in tightly closed containers in room temperature.

### 2.3. Physicochemical and Physical Analyses 

The substrates and the obtained biochars were analyzed for moisture content (by oven drying at 105 °C), organic matter (by incineration in a muffle furnace at 550 °C for 5 h and then calculate loss of weight based on certain assumptions) and ash (in accordance with PN-EN ISO 18122:2016-01 [78]). Substrates were analyzed for total carbon content (by Multi N/C, Analytkjena—the high-temperature incineration with detection IR [79]) and Kjeldahl nitrogen content (in accordance with PN-EN 16169:2012 [80]). pH measurement was made by placing 5 g of the sample in three, individual beakers and then adding distilled water to each of them (50 mL). The beakers were shaken for 10 min and then infiltrated. pH was measured by pH meter. Biochars derived at selected temperatures were analyzed for elemental composition, total organic carbon content, surface area, morphology and presence of surface functional groups. The CHNS elemental analysis was performed with the Thermo Scientific™ FLASH 2000 method of dynamic incineration (3–4 independent incineration). Total organic carbon content was indicated by analyzer TOC-5000A (made by Shimadzu, Kioto, Japan) with SSM 5000 attachment. TOC was indicated by high-temperature incineration (900 °C). Carbon dioxide was measured by infrared spectrometry and expressed as carbon. The BET surface area was indicated by the ASAP 2420. The ASAP 2420 analyzer (company Micromeritics, Norcross, GA, USA) measures single and multi-point specific surface areas and size and distribution of pore solid samples. The analytical techniques included scanning electron microscopy (SEM), which was used to examining the morphology of samples and chemical analysis (EDS). Surface functional groups were identified by Attenuated Total Reflectance Fourier transform infrared spectroscopy.

### 2.4. Statistical Analysis

The statistical analyses were conducted using IBM SPSS Statistics 26. The significance level was α = 0.05. To investigate the relationship of the type of substrate and the pyrolysis temperature with the parameters of the obtained biochar, an experimental study was conducted in a 3 × 4 intergroup plan. Independent variables were biomass type (3) and pyrolysis temperature (4). The dependent variables were the six parameters of the obtained biochar—pH, ash (ash content), N (nitrogen content), C (carbon content), H (hydrogen content) and BET (BET specific surface area). The results of this experiment were analyzed with a two-factor variance analysis for the intergroup plan in a 3 × 4 scheme. Post hoc testing was performed using the Bonferroni test.

## 3. Results and Discussion

### 3.1. Pyrolysis Process Yield

The implementation of the pyrolysis process in the temperature range of 400 °C to 700 °C produced a biochar yield in the range of 22.0 to 40.5%. The process yield fell with increasing process temperature (Table 2), as other studies have also demonstrated [50,81,82,83]. This dependence results from the decomposition of organic compounds as the process temperature grows [10]. A higher process yield at a lower temperature is more desirable, given the reduced costs [48].

The highest biochar yield from the substrates used in the study, reaching a level of 40.5%, was determined for the lowest temperature applied (400 °C), while for a temperature of 700 °C it fell within the range of 22.0 to 30.2%. Similar yield levels were found in other studies during the pyrolysis of plant substrates [51,60]. The authors of [60] found a decrease in the yields of solid pyrolysis products from 37.1% to less than 20% for a temperature increase in the range of 377–678 °C. The pyrolysis of pelletized sunflower husk produced the process yield of about 33.9–31.6% at temperatures in the range of 480 to 580 °C [51]. In the present study, the highest process yield among substrates was found for walnut shell, while the lowest one could be seen for beech wood chips. These results confirm the dependence of the pyrolysis process yields on the type of substrate subjected to conversion, as also demonstrated by [45,50,84]. This dependence is very conspicuous even within individual wood types. In their research, the authors of [48] found a significantly higher pyrolysis process yield for oak wood than the one in the research on beech wood considered above. The most effective process in the present study turned out to be the pyrolytic conversion of walnut shell carried out at a temperature of 400 °C. Other authors [1,85] have come to similar conclusions, demonstrating that the pyrolysis temperature within the range of 350 to 400 °C is the optimum temperature enabling the yield at a level of 45–35% to be achieved in the biochar production from plant crop residues.

The yield of the pyrolysis of plant wastes carried out at a temperature of 400 °C was much lower than that in the case of the pyrolytic conversion of wastes of animal origin, e.g., poultry manure [50] or sewage sludge [12]. In their research, the authors of [50] achieved a 51.52% biochar yield of the pyrolysis of poultry manure at a temperature of 400 °C, while the authors of [12] attained a 49% biochar yield. The differences in biochar yields from different wastes results from their different chemical characteristics related, among others, to ash content [50] or lignin content [86].

### 3.2. Chemical Characteristics of the Biochar Produced

The biochars produced in the pyrolysis of plant substrates, denoted as BWRS (biochar from wheat and rye straw), BWS (biochar from walnut shell) and BWC (biochar from beech wood chips) were subjected to a physico-chemical analysis, including the determination of the contents of organic matter (OM), ash, nitrogen (N), carbon (C), hydrogen (H), sulphur (S), total organic carbon (TOC in dry mass) and pH. Table 2 shows the results of the analysis.

The biochars produced from wheat and rye straw were alkaline as their pH fell within the range of 8.31 to 10.13, making them a potential agent to reduce soil acidity [48]. The biochars produced from walnut shell and beech wood chips were characterized by lower pH values, falling, respectively, within the ranges of 5.91 to 9.86 and 4.42 to 7.85. The determined pH values of biochars produced from different plant substrates confirmed the tendency for pH values to grow with increasing temperature of the pyrolysis process and the differentiation of pH values depending on the type of substrate, as presented by other authors [45,48,50]. The changes in the pH of biochars accompanying temperature changes are caused by the production of organic acids and phenolic compounds from the decomposition of cellulose and hemicellulose at low temperatures, and the release of volatile substances (composed of acidic functional groups) [10] and increased ash content (the presence of alkaline mineral compounds) at higher temperatures [8].

As a result of the analyses of the dependent pH variable a statistically significant main effect of the type of substrate variable was achieved. The post hoc tests of the analyzed variable showed statistically significant differences in each variant of substrate comparisons. This means that the BWRS biochar has the highest pH, followed by the BWS biochar, and the BWC biochar is characterized by the lowest pH. The main effect of the pyrolysis temperature variable was also statistically significant. The post hoc tests also proved to be statistically significant in each variant of pyrolysis temperature comparisons with the pH variable. This result indicates a trend where a lower pyrolysis temperature is associated with a lower pH of the produced biochar, the lowest pH is achieved at 400 °C and the highest at 700 °C.

A statistically significant effect of the interaction of the two factors on the pH level of the produced biochar was also achieved. Analysis of simple effects revealed significant statistical differences in all comparisons made in pairs, thus revealing a trend in which pyrolysis temperature is associated with pH levels in biochar groups. This result means that the increase in pyrolysis temperature is associated with a higher pH level among all three types of produced biochar. The results are presented in Table 3 and Table 4.

The analysis of the ash content in the biochars showed the highest ash content in the BWRS biochars. This content fell within the range of 10.55% to 14.44%. The remaining biochars had a lower ash content in the range of 1.06–3.00% (BWS) and 1.18–1.61% (BWC). Biochar generated at higher pyrolysis temperature had higher ash content. This tendency was also observed by other researchers [50,60]. The ash content in biochars of plant origin is lower than in the case of biochars produced from fecal substrates [50,60].

Calculations involving ash dependent variable revealed the statistically significant main effect of the type of substrate variable. Comparisons in pairs, post hoc, of the analyzed variable showed statistically significant differences between all types of biomass. The difference in ash variable level between BWS and BWC biochar is very low and the confidence intervals in the post hoc test are approaching zero. Further analysis of contrasts did not show statistically significant differences in ash levels in the BWC biochar group. It can, therefore, be assumed that, despite the significant result of the a posteriori analysis, the a priori result prejudges the absence of differences between the BWS and BWC biochar groups. Thus, BWRS biochar has a higher ash content compared to BWS and BWC biochar, but there are no differences in the ash levels between BWS and BWC biochar. The main effect of pyrolysis temperature variable has also proved to be statistically significant. The post hoc tests were statistically significant in all comparison in pairs groups, except for the comparison of the 600 °C temperature with 700 °C. This result reveals a trend in which higher temperatures are associated with more ash in the tested biochar, but there are no differences in ash levels during pyrolysis at 600 °C and 700 °C.

Statistically significant was also the effect of the interaction of both factors on ash levels in produced biochar. A post hoc analysis of the simple effects for the BWRS biochar group showed statistically significant differences in ash levels between all pyrolysis temperature levels except the 600 °C with 700 °C comparison. In the BWS biochar group, differences in ash levels were statistically significant for all comparisons in pairs, except for 500 °C with 600 °C and 600 °C with 700 °C. In the BWC biochar group, no statistically significant differences between pyrolysis temperature levels were revealed. This means that a higher temperature is associated with more ash in BWRS biochar, although this association fades above 600 °C. Similarly, the BWS biochar has a higher amount of ash with a higher temperature, but it is not observed to differ at temperatures of 500 °C and 600 °C as well as 600 °C and 700 °C. The temperature is unrelated to the level of ash in BWC biochar. The details of the analyses carried out are set out in Table 3 and Table 4.

The organic carbon content in the biochars produced fell within the range of 62.5% to 87.0%. Generally organic carbon content was found to grow with increasing temperature in the biochars produced from all the substrates. Taking into account the requirements of the European Biochar Certificate [87] for the organic carbon content in biochars applied in agriculture, all the biochars produced met the condition that the organic carbon content should be higher than 50% dry weight. 

An elemental analysis to determine the carbon content in the biochars demonstrated that the content of this element increased as the temperature of the pyrolysis process increased. It was only in the case of the biochars produced from the walnut shell substrate that the carbon content did not clearly increase. The highest carbon content was determined in the case of the biochars produced from beech wood chips. In addition to the ion exchange capacity, this property helps plants take up nutrients [88], which is significant for the use of biochars for fertilizer production. 

The analysis of the dependent variable C did not show a statistically significant main effect of the type of substrate variable and the pyrolysis temperature variable. There was also no statistically significant effect of the interaction of the two factors on the amount of carbon in the produced biochar. It turns out that the biomass and pyrolysis temperature are not associated with the carbon content of the produced biochar. Table 3 and Table 4 show the details of the carried-out analyses.

The hydrogen content in all the samples of the biochars analyzed decreased with an increasing temperature of the pyrolysis process, as a result of chemical reactions unfolding during the substrate pyrolysis process (dehydration, loss of C-bound H atoms caused by thermal degradation) [21]). 

The main effect of the substrate type variable on the dependent variable H was statistically significant. The post hoc analyses revealed only one statistically significant difference between BWRS and BWC biochar, differences in hydrogen content between BWRS and BWS and BWS and BWC groups are statistically insignificant. The main effect of the pyrolysis temperature variable was also statistically significant. The post hoc analysis calculations of the tested variable revealed statistically significant differences in each variant of pyrolysis temperature comparisons regarding hydrogen content in biochar. This result indicates a trend in which a lower pyrolysis temperature is associated with more hydrogen in the tested biochar.

A statistically significant effect of the interaction of the two factors on the hydrogen content of the produced biochar was achieved. The simple effect calculations revealed statistically significant differences in all comparisons made in pairs, thus showing a trend in which pyrolysis temperature is associated with hydrogen content in biochar groups. This means that a lower pyrolysis temperature is associated with a higher hydrogen content among all three types of produced biochar. The results of the analyses are presented in Table 3 and Table 4.

The value of the H/C ratio decreased with an increasing pyrolysis temperature at which the biochars were produced. The decreasing H/C ratio indicates carbonization and increased aromaticity of the products derived [10,53]. In accordance with the requirements of [87], the H/C ratio should not exceed 0.7. This requirement was met by all the biochars produced. The aromaticity of biochars can indicate their stability and, accordingly, their greater carbon sequestration potential [88]. The composition affecting the stability of biochars [72]. 

An analysis of the nitrogen content in the biochars from walnut shell and beech wood chips did not demonstrate the presence of this element as a result of its low content in these substrates. The nitrogen content in the biochars from wheat and rye straw fell within the range of 0.28 to 0.98% and grew with an increasing process temperature, which might be caused by the presence of nitrogen compounds with a structure resilient to thermal degradation [10]. No sulfur was found in the biochars examined.

When analyzing the dependent variable N, the statistically significant main effect of the type of substrate variable was revealed. Comparisons in pairs (post hoc analysis) showed two statistically significant differences between biomass of the BWRS and the BWS type, as well as between BWRS and BWC, differences in nitrogen content level between the BWS and BWC groups are statistically insignificant. The mean nitrogen content of BWS and BWC biochar is 0, this result indicates that the BWRS biochar has a higher nitrogen content compared to zero amounts of this element in BWS and BWC biochar. The main effect of pyrolysis temperature variable has also proven to be statistically significant. The post hoc tests of the tested variable revealed statistically significant differences in each variant of pyrolysis temperature comparisons in terms of nitrogen content in biochar, except for differences between 400 °C and 500 °C biochar. This result means that higher temperatures are associated with more nitrogen in the tested biochar, but there are no noticeable differences between pyrolysis at 400 °C and pyrolysis at 500 °C.

The effect of interactions between the two factors on the nitrogen content of the produced biochar has turned out to be statistically significant. The analysis of simple effects revealed statistically significant differences only in the BWRS type biochar group, between all pairs of pyrolysis temperature variables, except for differences between biochar produced at 400 °C and 500 °C. Other temperature differences in the BWS and BWC biomass groups were statistically insignificant, as their mean is 0. It appears, therefore, that the increase in pyrolysis temperature is associated with a higher nitrogen content in the case of biochar of the BWRS type, while in other types of biochar it decreases to zero. The results are presented in Table 3 and Table 4.

### 3.3. BET Specific Surface Area and Microstructure of Biochars

The specific surface area (BET) of the biochars produced from plant origin substrates can range from 1 to 363 m^2^·g^−1^ (Table 5). This diversity depends on the type and origin of a substrate which was used for production of biochar. The literature reports many studies on the effect of temperature on various properties of biochars, including the specific surface area [3,50,57,58,59,89]. For example, this was confirmed by the study on beech wood chips and walnut shell where the increase in temperature of pyrolysis led to the increase in specific surface area. Larger specific surface areas determined for products of plant origin derived at higher temperatures might be caused by the decomposition of lignocelullose and the evaporation of mineral inorganic substances [3,90]. Giudicianni et al. (2017) [60] achieved a wide specific surface range for biochars produced from plant biomass, which, however, had been earlier subject to the impact of steam. In this context, the authors of [60] emphasized a positive effect of steam which contributed to the desorption and removal of volatile substances, in contrast to the pyrolysis in a nitrogen atmosphere which generated products with lower porosity due to the deposition of carbonaceous material inside biochar pores. This phenomenon could also occur in the case of biochars analyzed in this study. 

Among the plant substrates, beech wood chips proved to be the best material for the production of biochars with the most developed specific surface area. As a result of their production at temperatures of 600 °C and 700 °C, they had a specific surface area of more than 300 m^2^·g^−1^. As a result of the pyrolysis temperature shift from 500 °C to 600 °C, the specific surface area grew by almost 250%. In their research, Liu et al. (2010) [44] determined the specific surface area of the biochar produced from pine wood at a temperature of 700 °C at a much lower level of 29 m^2^·g^−1^, which, as the authors explained, could have been an effect of the softening and melting of pine wood components. However, Efika et al. (2018) [7] determined the specific surface area of the biochars produced from pine sawdust pellets at a temperature of 800 °C, with a heating rate of 5 °C min^−1^, at quite a high level of 219 m^2^·g^−1^. In the present study, the specific surface area of the biochars produced from beech wood chips at a temperature of 400 °C, was 2 m^2^·g^−1^, i.e., similar to the one determined by the authors of [47] for the biochar derived, among others, from oak wood at temperatures of 400 and 450 °C. As reported by Srinivasan et al. (2015) [88], the biochar sorption capacity is proportional to the specific surface area and aromaticity, and biochars with a large specific surface area (>400 m^2^·g^−1^) can be very effective adsorbents. Among the analyzed biochars, only the biochar produced from beech wood chips at a temperature of 700 °C had the specific surface area closest to the area cited above. The other biochars had a much smaller BET specific surface area. 

An analysis of the BET specific surface area of the biochars produced from walnut shell demonstrated that at a temperature of 600 °C the specific surface area of the biochars was about 164 m^2^·g^−1^, and it did not exceed this value when the temperature rose to 700 °C. Perhaps in the case of this substrate, an even higher pyrolysis temperature needs to be applied to increase the specific surface area. Analyzing the effect of temperature on the specific surface area of biochars, Hung et al. (2017) [3] found a rapid increase in the BET specific surface area from 6 m^2^·g^−1^ at a temperature of 700 °C to 102 m^2^·g^−1^ when the pyrolysis process was carried out at a temperature of 800 °C. Ahmad et al. (2012) [45] determined a much larger specific surface area in the case of biochars produced from soybean stover and peanut shell at temperatures of 300 and 700 °C. They found specific surface areas, respectively, in the range of 5 to 420 m^2^ g^−1^ (soybean stover), and from 3 to 448 m^2^ g^−1^ (peanut shell). In the case of the biochars produced from wheat and rye straw, the biochars produced at a temperature of temperature 500 °C had the largest specific surface area, i.e., about 12 m^2^·g^1^. In this case, an increase in the biochar production process temperature did not cause the specific surface area to grow. At temperatures of 600 °C and 700 °C, the BET specific surface area was found to diminish, respectively, to 8 m^2^·g^−1^ and 3 m^2^·g^−1^. The lack of correlation between the specific surface area of biochars with increasing temperature might be caused by the heterogeneity of the substrate which was a mixture of wheat and rye straw. The authors of [53] found a much larger BET specific surface area for biochars produced from maize straw at a temperature of 600 °C, i.e., about 353 m^2^·g^−1^ (with 17.36% ash content). 

An analysis involving the BET dependent variable showed a statistically significant main effect of the type of substrate variable. Comparisons in pairs through post hoc analysis showed statistically significant differences between all types of the used biomass. This means that the BWRS biochar has a smaller BET specific surface area compared to BWS and BWC biochar, and the BWS biochar has a smaller BET specific surface area compared to the BWC biochar. The main effect of the pyrolysis temperature variable has also proved to be statistically significant. The post hoc tests revealed statistically significant differences in all groups of comparisons in pairs. This result reveals a trend in which a higher pyrolysis temperature is associated with a larger BET specific surface area of the tested biochar.

The effect of the interaction of the two independent variables on the level of the BET dependent variable of the produced biochar turned out to be statistically significant. The post hoc analysis of the simple effects for the BWRS biochar group also showed two statistically significant differences in the level of the BET variable in comparison of the pyrolysis temperature of 400 °C with 500 °C and 500 °C with 700 °C. Other tests of differences were revealed to be statistically insignificant. In the BWS biochar groups, differences in the BET variable level were statistically significant for all comparisons in pairs. Likewise, in the BWC biochar group, statistically significant differences between all pyrolysis temperature levels have been revealed. This result indicates a trend in which a higher pyrolysis temperature is associated with a larger BET specific surface area for the BWS and BWC biochar types. This trend is also present for BWRS biochar to a lesser degree. For BWS biochar, this trend reverses at 600 °C and 700 °C. The details of the carried-out analyses are shown in Table 3 and Table 4.

Activated carbon has the specific surface area of up to 1000 m^2^·g^−1^; therefore, it seems necessary to apply activation processes in order to attain the large specific surface area of biochars [46,73].

**Figure 2:**
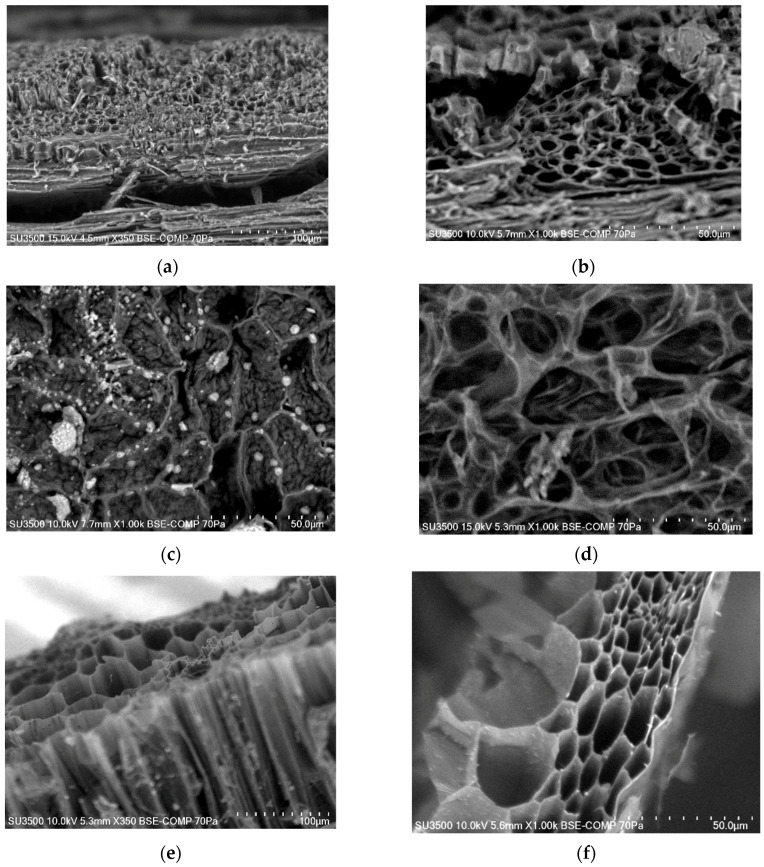
Microstructure of biochars: (**a**) BWC_400_; (**b**) BWC_700_; (**c**) BWS_400_; (**d**) BWS_700_; (**e**) BWRS_500_; (**f**) BWRS_700_.

Figure 2 shows the microstructure of the biochars produced. In the photos, voids and macropores can be seen. A characteristic system resembling a honeycomb, which can reflect the carbon skeleton of the capillary biological structure of the lignocellulosic feedstock, can also be seen [8]. In the case of biochars produced from beech wood chips and walnut shell, a difference can be seen between the microstructure of the biochars produced at a temperature of 400 °C (Figure 2a,c) and at a temperature of 700 °C (Figure 2b,d). The higher-temperature biochars are characterized by a better developed surface area with visible macropores. 

**Figure 3:**
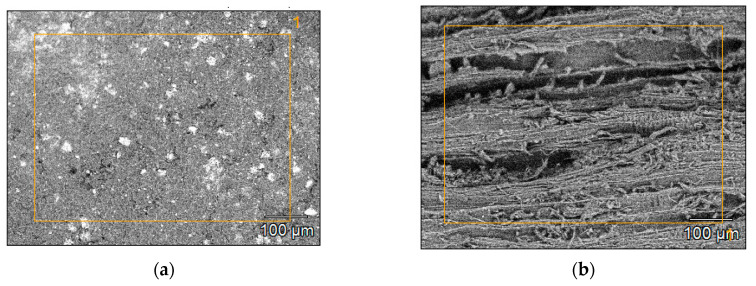

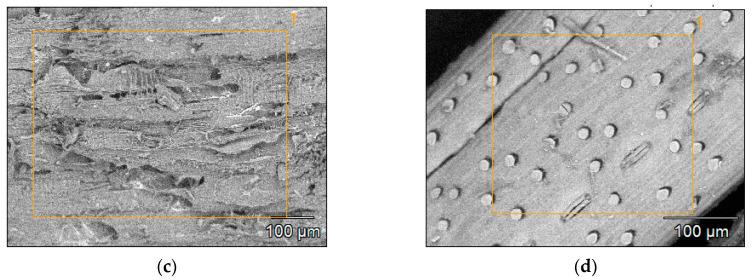
Surfaces of samples, with marked areas for which the elemental analysis was performed: (**a**) BWS_600_; (**b**) BWC_400_; (**c**) BWC_500_; (**d**) BWRS_600_.

Figure 2c shows the microstructure of the biochars produced from walnut shell at a temperature of 400 °C. Numerous, irregularly distributed lighter spots can be seen in the photo. In addition to carbon and oxygen, the EDS analysis of these areas (Figure 3a) demonstrated the presence of such elements as: Na, Mg, Al, Si, P, S, K, Ca and Fe. In several cases, among the elements analyzed, the presence of N, Cl, Ir, F, Ti, Ba and Pt was also determined. Figure 2a shows the microstructure of BWC_400_. In addition to carbon and oxygen, the EDS analysis of these biochars (Figure 3b) also demonstrated the presence of: Al, K and Ca. In addition, Mg appeared in BWC_500_ biochars (Figure 3c) as well as in Si in BWC_600_ i BWC_700_ biochars. Figure 2e,f show the microstructure of the biochars produced from wheat and rye straw at temperatures, respectively, of 500 °C and 700 °C. The microstructures of these biochars were very similar. BWRS_500_ biochars were characterized by the largest specific surface area of about 12 m^2^·g^−1^, while the biochars derived at a temperature of 700 °C had the smallest surface area, which was still regarded as small, at about 3 m^2^·g^−1^. The EDS analysis of the biochars produced from wheat and rye straw at a temperature of 600 °C (Figure 3d) demonstrated the presence of C, O, Mg, Al, Si, S, K, Ca and N (with the presence of the latter confirmed by the elemental analysis). 

### 3.4. Functional Groups in Biochars

The presence of specific types of surface functional groups in biochars can indicate the potential possibilities of their use, among others, in the processes of removing different contaminants from the environment, e.g., heavy metals from water or wastewater [13]. The intensity of surface functional groups was distinctly lower in the case of the biochars produced at a pyrolysis temperature of 700 °C than it was in the case of those derived at a temperature of 400 °C.

**Figure 4:**
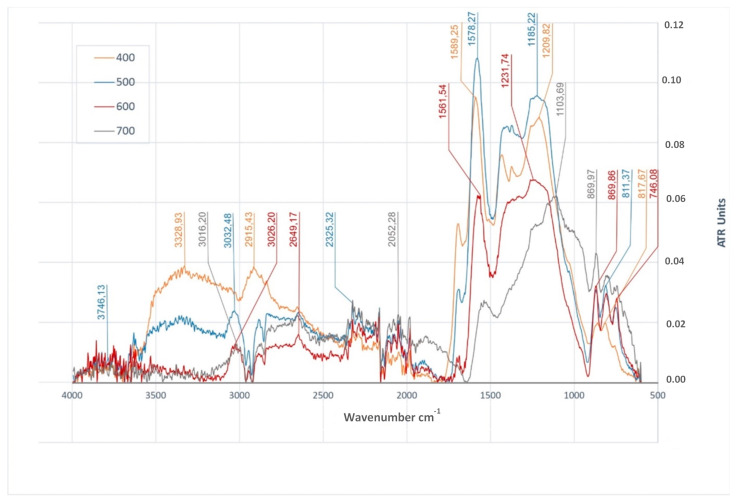
FTIR/ATR spectra of biochars produced at different temperatures (400 °C, 500 °C, 600 °C and 700 °C) from beech wood chips.

The analysis of the FTIR spectra (Figure 4) of biochar produced from beech wood chips (BWC) demonstrated the presence of stretching vibrations of O–H bonds (for wavelengths of 3328 cm^−1^), only in the case of biochar derived at a temperature of 400 °C. A similar situation was found in the case of the bending vibrations of O–H bonds, the presence of which was found for wavelengths of 1209 cm^−1^, only in the case of BWC_400_. The absence of stretching and bending vibrations of O–H bonds (or their lesser intensity) in the remaining samples of biochar from beech wood chips was probably caused, among others, by dehydration of the material and the loss of volatile substances, since OH groups are unstable at higher temperatures. A signal corresponding to stretching vibrations of C–H bonds (i.e., CH_2_ and CH_3_), present in aliphatic compounds [49], was observed in the case of biochar produced at any temperature (400, 500, 600, 700 °C) in the range of approximately 3032–2915 cm^−1^ of the FTIR spectrum (Figure 4). In the study reported in [48], aliphatic functional groups, which could transform into aromatic structures resulting in a greater presence of phenolic functional groups (ethers) (with vibrations for wavelengths of 1000 to 1250 cm^−1^), were not determined in biochar produced at temperatures of 600 °C and more. This can result in the decreased ability of biochar to adsorb nutrients, which stems from the presence of acidic functional groups [50]. In the case of samples of the tested biochar (BWC), the intensity of signals corresponding to the vibrations in question had a tendency to fall with increasing temperature, most likely due to the breaking of relatively weak C–H bonds [10]. The strongest signal corresponding to those vibrations was observed in the case of biochar produced at a temperature of 400 °C. The presence of CH groups in the analyzed FTIR spectra additionally confirmed the presence of bands in the range of 1470–1397 cm^−1^, and this signal was attributed to the bending modes of these groups [10]. Signals corresponding to the vibrations in the range of approximately 1430 cm^−1^ could be attributed to the bending vibrations of C = C and/or saturated C–H in lignin and carbohydrates [4]. An increase in the pyrolysis temperature activated the processes of the conversion of organic components (lignin, cellulose, and hemicellulose) and the release of volatile substances [10]. Therefore, the absence of signals in the range mentioned above might indicate the degradation of lignin, which diminished the availability of any carbon, as such a situation took place in the case of BWC_700_. The analysis of FTIR spectra (Figure 4) of BWC biochar demonstrated the presence of bands of the stretching vibrations of C=O bonds (in the range of approximately 1700 cm^−1^) in samples derived at temperatures of 400 °C, 500 °C, and 600 °C. The intensity of these signals fell with the increasing of the temperature. The occurrence of the vibrations of this type in the range of approximately 1700 cm^−1^ might indicate the presence of ketones, quinones, and/or carboxylic carbon [8]. In the analyzed FTIR spectra (Figure 4) of BWC biochar (from the entire temperature range), signals coming from the stretching vibrations of C=C bonds in the range of approximately 1594–1540 cm^−1^ were observed. The intensity of these signals fell with an increasing pyrolysis temperature. A pyrolysis temperature of 600 °C or higher could cause the breaking of many double C=C bonds, resulting in a decrease in the content of structures with C=C bonds in the biochar [48]. The signals observed, corresponding to the wave number in the range of approximately 1576 cm^−1^, might be related to the vibrations of the C=C grouping in an aromatic ring, coupled with the C=O carbonyl group [91]; this could indicate the generation of products with their structure containing an organooxygen structural grouping coupled with double C=C bonds in aromatic rings. In the case of FTIR spectra (Figure 4) of BWC biochar, produced at temperatures 500 °C, 600 °C, and 700 °C, bands attributed to the stretching modes of the C-O bonds were observed (in the range of approximately 1231–1005 cm^−1^), where their presence might suggest the emergence of aromatic ethers through the integration of oxygen atoms into cyclical carbon structures [91]. On the other hand, the absorption bands in the range of approximately 1050–850 cm^−1^ could be attributed to the stretching modes of C–O and the bending modes of O–H in alcohols, phenols, ethers, and esters [91]. The signals observed in the spectra of BWC_600_ and BWC_700_ biochar were the most intense of all the signals present in the FTIR spectra of that biochar. When combined with the largest specific surface area of the biochar produced from beech wood chips as described earlier, this feature may indicate the greatest potential of that biochar for the sorption of different types of contaminants. The presence of oxygen-containing functional groups on the surface of biochar is very desirable, mostly in the context of the application of biochar to immobilize metals. These groups are of large importance for the formation of organometallic complexes immobilizing heavy metals in soil (Pb (II), Cu (II), Ni (II) and Cd (II)) [9,41,92]. The analysis of the FTIR spectra of BWC biochar demonstrated the presence of the bending vibrations of =C–H bonds in the range of approximately 874–98 cm^−1^, which might indicate the presence of polycyclic aromatic structures [8]. 

**Figure 5:**
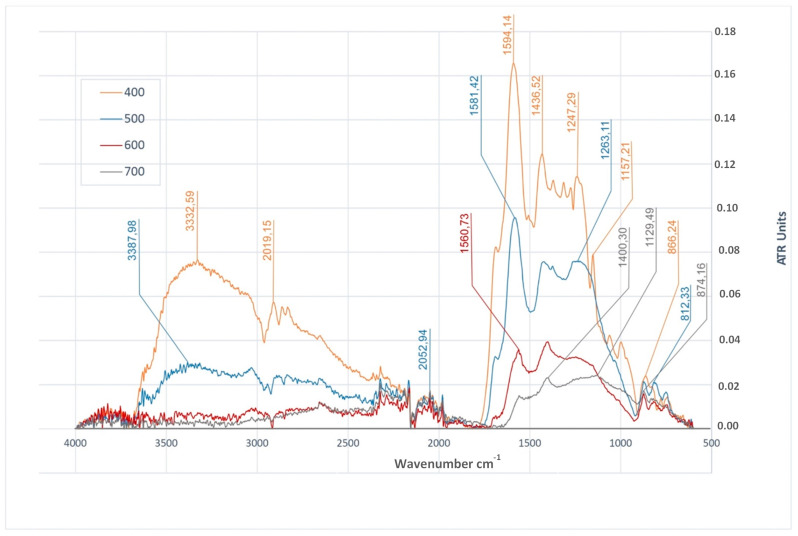
FTIR/ATR spectra of biochars produced at different temperatures (400 °C, 500 °C, 600 °C and 700 °C) from walnut shell.

In the case of the biochar produced from walnut shell (BWS), the analysis of FTIR spectra (Figure 5) demonstrated the presence of stretching vibrations of O–H bonds in products obtained at temperatures of 400 °C and 500 °C. These vibrations were observed for wavelengths of 3332 cm^−1^ and 3387 cm^−1^, respectively. A similar situation was found in the case of the bending vibrations of O–H bonds, the presence of which was found for wavelengths of 1247 cm^−1^ and 1263 cm^−1^, respectively, in BWS_400_ and BWS_500_ biochar. A group of bands attributed to the stretching modes of the C–H groups was observed in the case of BWS_400_, BWS_500_, and BWS_600_ biochar in the range of approximately 3050–2915 cm^−1^ of the FTIR spectrum (Figure 5). The analysis of FTIR spectra of samples of BWS biochar has demonstrated the presence of stretching vibrations of C=O bonds in the case of biochar produced at the temperatures of 400 °C and 500 °C. This might be caused, among others, by the presence of juglone—a substance present in walnut shell which is an organic chemical compound from the group of quinone dyes. In all the analyzed FTIR spectra (Figure 5) of the studied BWS biochar, signals coming from the stretching vibrations of C=C bonds (in the range of approximately 1594–1540 cm^−1^) were observed, the intensity of which was decreasing with the increase of the pyrolysis temperature. Bands assigned to stretching modes of C–O bonds were observed in the FTIR spectra of BWS biochar samples (in the range of approximately 1231–1005 cm^−1^). These vibrations were determined in all the biochar produced from walnut shell, except for BWS_500_, but this have been caused by the hampered analysis of its spectrum. The analysis of the FTIR spectra of BWS biochar (Figure 5) demonstrated the presence of the bending vibrations of =C-H bonds in the range of approximately 874–98 cm^−1^.

**Figure 6:**
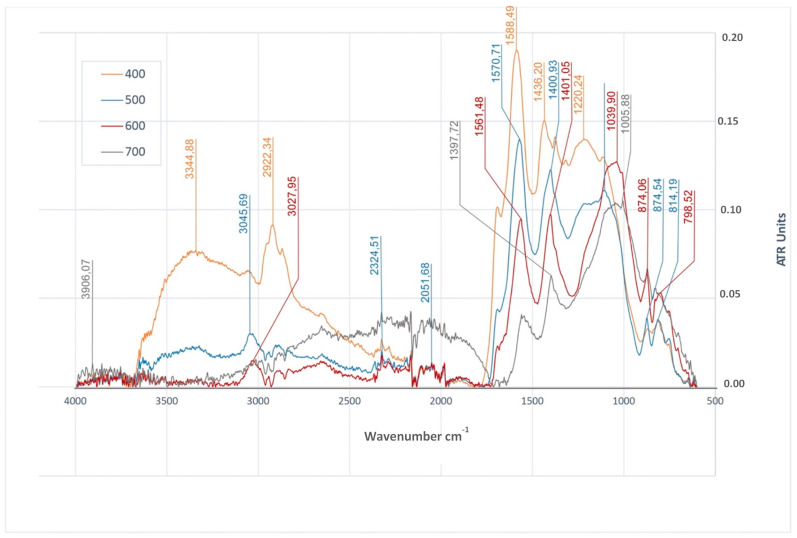
FTIR/ATR spectra of biochars produced at different temperatures (400 °C, 500 °C, 600°C and 700 °C) from wheat and rye straw.

The analysis of FTIR spectra of biochar produced from wheat and rye straw (Figure 6) demonstrated the presence of stretching vibrations of O–H bonds in the biochar derived at temperatures of 400 °C and 500 °C, respectively for wavelengths of 3344 cm^−1^ and 3330 cm^−1^. In these samples, the presence of bending vibrations of O–H bonds was also observed for wavelengths of 1220 cm^−1^ and about 1200 cm^−1^ in BWRS_400_ and BWRS_500_ biochar. A group of bands assigned to stretching modes of C–H groups in the case of BWRS_400_, BWRS_500,_ and BWRS_600_ biochar were observed in the range of approximately 3045–2922 cm^−1^ of the FTIR spectrum. The analysis of FTIR spectra (Figure 6) has also demonstrated the presence of bands of the stretching vibrations of C=O bonds (in the range of approximately 1700 cm^−1^) in samples derived at temperatures of 400 °C, 500 °C, and 600 °C. The intensity of these signals fell with the increasing temperature. In the analyzed FTIR spectra of all BWRS biochar, signals coming from stretching vibrations of C=C bonds in the range of approximately 1594–1540 cm^−1^ were observed. The intensity of these signals also fell with the increasing pyrolysis temperature. Bands assigned to stretching modes of C–O bonds were observed in the FTIR spectra of BWRS biochar samples in the range of approximately 1231–1005 cm^−1^. The bands corresponding to these vibrations were determined in the samples of all the biochar from wheat and rye straw and in the case of the biochar produced at temperatures of 600 °C and 700 °C. These signals were the most intense of all the signals present in the spectra of the biochar produced at a given temperature. In the case of biochar with small specific surface area, this property can be compensated for by the presence of C–O groups and have a positive effect on its sorption properties [88]. In the case of BWRS biochar, the analysis of the FTIR spectra has also demonstrated the presence of bending vibrations of =C-H bonds in the range of approximately 874–98 cm^−1^ indicating the presence of polycyclic aromatic structures. 

It has been shown, inter alia, the influence of the type of precursor used in the production of biochar on the presence of surface functional groups. The presence of intense stretching vibrations of C-O bonds, having a potential impact on the sorption capacity of biochars, was determined in the FTIR spectra of BWC600 and BWC700 biochars, this feature, combined with the large BET surface area, may affect the sorption potential of these biochars. The presence of this type of high-intensity vibrations was also observed in the spectra of biochar BWRS600 and BWRS700. This can compensate for the low BET surface value and play an important role when using these biochars in sorption processes for organic and inorganic compounds.

Table 6 collects data on spectral characteristics, their assignment to the functional groups of the biochar surface.

## 4. Conclusions

The pyrolytic conversion (in the temperature range of 400 °C–700 °C) of biodegradable wastes of plant origin may produce biochars with diversified properties. The present study found a clear effect of pyrolysis process temperatures on the yield and selected properties of biochars. The biochar production yield was found to diminish with increasing temperature. In terms of the biochar production yield, walnut shell proved to be the most attractive substrate. Temperature changes affected the chemical composition of the products derived. With increasing temperature, the content of carbon in biochars also increased but hydrogen content decreased. This resulted in lower H/C ratio. Increased pH and TOC values were also determined. Another parameter which demonstrated a growing tendency with increasing temperature was the BET specific surface area (except for BWRS). An increase in the pyrolysis temperature also caused a decrease in the diversity and intensity of the surface functional groups of biochars.

The alkaline character of biochars produced at higher temperatures makes them desirable materials to reduce soil acidity. High TOC content also confirms the potential for the use of biochars in agriculture. The increased carbonization and aromaticity of biochars produced at higher temperatures may determine their stability and carbon sequestration potential. The presence of oxygen-containing functional groups (the ability to form complexes) creates the potential opportunities for using such biochars as materials immobilizing selected heavy metals. The well-developed BET surface area and the presence of specific functional groups on the surface of biochars from beech wood chips may determine their adsorption capacity. Biochars produced at higher temperatures, poorer in carboxyl and hydroxyl groups, may show lesser capacity to adsorb N-NH_4_. 

The small specific surface area can be compensated for by the presence of specific functional groups and have a positive effect on their sorption properties. Therefore, the use of the biochars produced in the process of sorption of specific components, e.g., heavy metals or ammonia nitrogen, requires further research and possible modification of their properties in physical or chemical processes. 

It is planned to continue the research aimed at checking the effectiveness of the use of the tested biochars in the sorption of ammonium nitrogen and phosphorus from the leachate produced during the treatment of sewage sludge or municipal waste, and to reduce ammonia emissions from gases emitted during composting.

## Figures and Tables

**Table 1:** Selected properties of substrates for biochar production.

	MC%	OM%_dm_	Ash%_dm_	pH_H2O_	N_K_%	TC%_dm_
WRS	11.96 ± 1.52	96.08 ± 1.75	3.91 ± 1.75	7.05	0.507	44.260
WS	13.38 ± 0.17	98.94 ± 0.10	1.06 ± 0.10	4.98	0.299	49.923
WC	7.19 ± 0.04	99.34 ± 0.41	0.66 ± 0.41	5.11	0.213	50.101

**Table 2:** Biochar production yields at different temperatures and properties of the biochars produced.

Type of Biochar	Yield	pH_H2O_	OM	Ash	N	C	H	S	TOC	H/C
	%		% d.m.							
BWRS_400_	34.4	8.35 ± 0.08	89.45 ± 0.45	10.55 ± 0.45	0.28 ± 0.07	69.11 ± 0.69	3.87 ± 0.04	-	69.0	0.67
BWRS_500_	29.5	9.52 ± 0.04	88.36 ± 0.50	11,64 ± 0.50	0.32 ± 0.05	74.40 ± 0.13	2.97 ± 0.01	-	69.9	0.48
BWRS_600_	29.1	9.89 ± 0.01	86.12 ± 0.22	13.88 ± 0.22	0.65 ± 0.01	77.32 ± 0.12	2.33 ±0.13	-	76.5	0.36
BWRS_700_	25.5	10.10 ± 0.11	85.56 ± 0.61	14.44 ± 0.61	0.98 ± 0.12	78.48 ± 0.19	1.45 ± 0.12	-	77.9	0.22
BWS_400_	40.5	5.98 ± 0.06	98.94 ± 0.28	1.06 ± 0.28	-	79.40 ± 1.16	3.96 ± 0.06	-	79.2	0.60
BWS_500_	34.2	6.90 ± 0.05	98.05 ± 0.09	1.95 ± 0.09	-	78.03 ± 5.93	3.22 ± 0.03	-	77.3	0.49
BWS_600_	31.4	7.37 ± 0.01	97.38 ± 0.17	2.62 ± 0.17	-	63.24 ± 0.95	2.20 ± 0.02	-	62.5	0.42
BWS_700_	30.2	9.85 ± 0.01	97.00 ± 0.31	3.00 ± 0.31	-	77.38 ± 0.67	1.38 ± 0.05	-	76.4	0.21
BWC_400_	31.7	4.45 ± 0.04	98.65 ± 0.08	1.35 ± 0.08	-	76.65 ± 0.35	4.20 ± 0.11	-	75.6	0.66
BWC_500_	25.1	5.18 ± 0.03	98.81 ± 0.06	1.18 ± 0.06	-	82.39 ± 0.81	3.02 ± 0.01	-	81.9	0.44
BWC_600_	23.2	6.57 ± 0.04	98.51 ± 0.05	1.49 ± 0.05	-	88.03 ± 6.61	2.53 ± 0.32	-	87.0	0.34
BWC_700_	22.0	7.82 ± 0.03	98.37 ± 0.08	1.61 ± 0.08	-	84.61 ± 10.03	1.47 ± 0.14	-	84.0	0.21

**Table 3:** The results of the two-factor analysis of variance testing the diversity of biochar properties in terms of the type of substrate and pyrolysis temperature.

	pH	Ash	N	C	H	BET
Main effect—type of substrate	13,749.03 ***	5134.79 ***	681.22 ***	0.08	5.08 *	12,447.77 ***
Main effect—temperature	5486.10 ***	86.53 ***	58.53 ***	1.85	737.42 ***	8096.43 ***
Interactive effect—type of substrate * temperature	446.88 ***	24.27 ***	58.53 ***	2.16	3.56 *	2807.52 ***

* *p* < 0.05; *** *p* < 0.001.

**Table 4:** The mean and standard deviation of biochar properties in relation to the type of substrate and pyrolysis temperature.

		pH		Ash		N		C		H		BET	
		M	SD	M	SD	M	SD	M	SD	M	SD	M	SD
BWRS		9.47	0.71	12.63	1.71	0.56	0.30	74.83	3.79	2.66	0.93	7.41	3.94
BWS		7.53	1.50	2.16	0.79	0.00	0.00	74.51	7.33	2.69	1.03	81.79	80.23
BWC		6.01	1.35	1.41	0.18	0.00	0.00	76.53	24.94	2.81	1.04	191.51	156.48
400		6.26	1.70	4.32	4.68	0.09	0.14	66.54	25.38	4.01	0.16	2.70	1.45
500		7.20	1.89	4.92	5.05	0.11	0.16	78.27	4.58	3.07	0.12	37.28	39.40
600		7.94	1.50	6.00	5.93	0.22	0.33	76.20	11.27	2.35	0.23	161.65	131.26
700		9.26	1.08	6.35	6.11	0.33	0.49	80.16	6.05	1.43	0.10	172.66	156.15
BWRS	400	8.35	0.08	10.55	0.45	0.28	0.07	69.11	0.69	3.87	0.04	4.52	0.06
	500	9.52	0.04	11.64	0.50	0.32	0.05	74.40	0.13	2.97	0.01	12.99	0.07
	600	9.89	0.01	13.88	0.22	0.65	0.01	77.32	0.12	2.33	0.13	8.71	0.05
	700	10.10	0.11	14.44	0.61	0.98	0.12	78.48	0.19	1.45	0.12	3.43	0.06
BWS	400	5.98	0.06	1.06	0.28	0.00	0.00	79.40	1.16	3.96	0.06	1.23	0.02
	500	6.90	0.05	1.95	0.09	0.00	0.00	78.03	5.93	3.22	0.03	9.11	0.13
	600	7.37	0.01	2.62	0.17	0.00	0.00	63.24	0.95	2.20	0.02	164.48	1.83
	700	9.85	0.01	3.00	0.31	0.00	0.00	77.38	0.67	1.38	0.05	152.35	4.00
BWC	400	4.45	0.04	1.35	0.08	0.00	0.00	51.10	44.26	4.20	0.11	2.36	0.05
	500	5.18	0.03	1.18	0.06	0.00	0.00	82.39	0.81	3.02	0.01	89.73	2.86
	600	6.57	0.04	1.49	0.05	0.00	0.00	88.03	6.61	2.53	0.32	311.77	4.04
	700	7.82	0.03	1.61	0.08	0.00	0.00	84.61	10.03	1.47	0.14	362.19	7.44

M—mean; SD—standard deviation.

**Table 5:** BET specific surface area of biochar produced at different temperatures.

Type of Biochar	400 °C	500 °C	600 °C	700 °C
	m^2^·g^−1^			
BWRS	4	12	8	3
BWS	1	9	164	152
BWC	2	89	311	363

**Table 6:** FTIR Peak assignment of characteristic vibrations observed for biochars produced at different temperatures.

FTIR Peak cm^−1^	Characteristic Vibrations	BWC				BWS				BWRS				References
		400 °C	500 °C	600 °C	700 °C	400 °C	500 °C	600 °C	700 °C	400 °C	500 °C	600 °C	700 °C	
3328–3387	O–H stretching	**+**	**−**	**−**	**−**	**+**	**+**	**−**	**−**	**+**	**+**	**−**	**−**	[8,33,36,48,71,88]
2915–3050	C–H stretching	**+**	**+**	**+**	**+**	**+**	**+**	**+**	**−**	**+**	**+**	**+**	**−**	[8,33,48,49,88,93]
1700	C=O stretching	**+**	**+**	**+**	**−**	**+**	**+**	**−**	**−**	**+**	**+**	**+**	**−**	[8,36,52,71,88]
1540–1594	C=C stretching	**+**	**+**	**+**	**+**	**+**	**+**	**+**	**+**	**+**	**+**	**+**	**+**	[8,33,36,48,71,88,91,93]
1397–1470	C–H deformation	**+**	**+**	**+**	**−**	**+**	**+**	**+**	**+**	**+**	**+**	**+**	**+**	[10,36,71]
1200–1290	O–H deformation	**+**	**−**	**−**	**−**	**+**	**+**	**−**	**−**	**+**	**+**	**−**	**−**	[8,93]
1005–1231	C–O stretching	**−**	**+**	**+**	**+**	**+**	**−**	**+**	**+**	**+**	**+**	**+**	**+**	[8,33,52,88,91]
746–874	=C–H bending	**+**	**+**	**+**	**+**	**+**	**+**	**+**	**+**	**+**	**+**	**+**	**−**	[8,36,48,52,88]

**^+^** observed; **^−^** not observed.

## Data Availability

Not applicable.
